# Supplementary material for: 4-Ethylacetophenone from Potato Plants Repels Phthorimaea operculella and Inhibits Oviposition: A Sustainable Management Strategy
Source: Insects. 2025 Apr 11;16(4):403. doi: 10.3390/insects16040403 (PMC12027768; doi:10.3390/insects16040403)
Supplement: Supplementary file 1 [file insects-16-00403-s001.zip › insects-3476958-supplementary.pdf]

Table S1

Table S1 Electrophysiological responses of *P. operculella* to compounds

Compounds	EAG relative response value（mV）				
	0.01 μg	0.1 μg	1 μg	10 μg	100 μg
4-hydroxy-4-methyl-2-pentanone	0.064 ± 0.130a	0.023 ± 0.063a	0.025 ± 0.109a	0.013 ± 0.040a	-0.008 ± 0.031a
Ethylbenzene	0.018 ± 0.105a	-0.010 ± 0.089a	0.061 ± 0.091a	0.033 ± 0.082a	0.034 ± 0.062a
Geranylacetone	0.076 ± 0.071b	-0.030 ± 0.099b	-0.046 ± 0.081a	0.076 ± 0.104b	0.087 ± 0.088b
4-ethylacetophenone	-0.009 ± 0.094b	0.027±0.132b	0.023 ± 0.123b	0.169 ± 0.097a	0.124 ± 0.075a
3-ethylacetophenone	0.056 ± 0.085b	0.017±0.068b	0.018 ± 0.044b	0.197 ± 0.112a	0.225 ± 0.118a

Note: Data in the table are mean ± standard error (SEM). Different lowercase letters labelled after the data in the same row indicate significant differences ($p < 0.05$).
